# ﻿*Camelliayangii* (Theaceae), a new species of tea plants (*Camellia* section *Thea*)

**DOI:** 10.3897/phytokeys.257.152000

**Published:** 2025-06-13

**Authors:** Dongwei Zhao

**Affiliations:** 1 Department of Forestry, College of Forestry, Central South University of Forestry and Technology, 498 Shaoshan South Road, Changsha, Hunan 410004, China Central South University of Forestry and Technology Changsha, Hunan China

**Keywords:** Beverage, diversity, genetic resources, Yunnan

## Abstract

Camelliasect.Thea contains plants of beverage sources with huge profits. Their natural germplasm resources have yet to be fully explored. Here, morphological, phenological and phylogenetic analyses were undertaken to reveal a new species of tea plants, *C.yangii* D.Wei Zhao. It is described with an illustration and photos of fresh characters provided. The new species is similar to *C.fangchengensis* and *C.ptilophylla* by the densely pubescent new branchlets, abaxial surface of leaves and pedicel, but differs from them in bearing a larger flower, fewer (3 vs. 5) but larger sepals, and the indumentum of the sepals. Molecular phylogenetic analysis using *RPB2* introns 11–15 and 23 and *waxy* suggests that it is a member of C.sect.Thea and its phylogenetically closely related species are *C.longissima* and *C.taliensis*. *Camelliayangii* has a later flowering phase compared with other taxa of C.sect.Thea that occurred or were planted nearby, so it cannot naturally hybridize with other tea plants. The new species bears a red or purplish red and densely pubescent terminal bud, which suggests it is a rare germplasm resource of tea plants. *Camelliayangii* is only known from a single extremely vulnerable population and strict conservation and asexual propagations are urgently needed to avoid extinction.

## ﻿Introduction

Tea (*Camelliasinensis* [L.] Kuntze) is the most widely cultivated commercial crop of *Camellia* L. (Theaceae). Its leaf buds, leaves, and young branches are manufactured to yield several kinds of beverages, such as black, green, and oolong tea (Zhao 2022). Tea and its relatives, the tea plants (C.sect.Thea [L.] Griff.), all can be used as beverage sources and their natural populations are the vital germplasm resources of current cultivars of tea (Zhao 2024).

Griffith (1854) established C.sect.Thea (Zhao et al. 2017; Zhao 2022). The section was subsequently revised by different taxonomists mainly based on morphological and phytochemical investigations, such as Dyer (1874), Cohen-Stuart (1916), Sealy (1958), Chang (1981a, 1984, 1998), and Ming (1992, 1999, 2000). Molecular phylogenetic analyses using nuclear DNA regions supported the monophyly of C.sect.Thea (Xiao and Parks 2003; Vijayan et al. 2009; Zhao et al. 2023). By contrast, phylogenomic analyses based on the complete plastid genome did not suggest that the samples of C.sect.Thea formed a monophyletic group (Yang et al. 2013; Yu et al. 2017; Yan et al. 2021). However, the monophyly of the section was generally supported in the phylogenomic investigations using data from transcriptomes of *Camellia* (Wu et al. 2022; Zan et al. 2023). Based on the phylogenetic analyses of biparentally inherited nuclear DNA rather than maternally inherited plastid DNA and morphological and phytochemical studies, Zhao (2024) revised C.sect.Thea comprised 11 natural species and seven infraspecific taxa, including a new member, *C.longissima* Hung T.Chang & S.Ye Liang. They all bear pedicellate flowers with 2–4 caducous bracteoles, persistent sepals, glabrous stamens, an apically 3–5-lobed style, and glabrous seeds. All these tea plants can be found in China. Three of them, including C.sinensisvar.assamica (Royle ex Hook.) Steenis, C.sinensisvar.pubilimba Hung T.Chang, and *C.taliensis* (W.W.Sm.) Melch. also occur in Indochina (Zhao 2024).

During several fieldworks in Yunnan, China in 2023, a special plant of *Camellia* was found in the forest of Malipo County. Subsequent morphological, phenological and phylogenetic analyses suggested that it was a distinct species of C.sect.Thea. The new tea plant is described below.

## ﻿Materials and methods

### ﻿Field investigations and sampling

Several fieldworks were undertaken to collect the specimens that bore flowers and/or fruits for the interested new species and other species of *Camellia* nearby. Photos of the habitat, habit, and fresh characters of the vegetative and propagative organs of the plants were taken. The flowering phases of different species were recorded. Clean and young leaves were collected and rapidly dried in silica gel, then stored at −30 °C for total DNA extraction. Voucher specimens were collected and deposited at the herbaria listed in Suppl. material 1. Samples were identified using a narrow circumscription based on Chang (1998) and the protologues.

### ﻿Morphological and phylogenetic analyses

Morphological characters of the species were measured, described, and compared based on photos of living plants and specimens collected or examined at herbaria (acronyms following Thiers [2025], continuously updated) BM, CSFI, E, GXFI, GXMI, HITBC, IBK, IBSC, K, KUN, P, PE and SYS. Articles of publishing a new species in the Shenzhen Code (Turland et al. 2018) were complied with.

Total genomic DNA extraction and subsequent molecular phylogenetic analyses with three nuclear regions, including *RPB2* introns *11–15* and intron 23 and *waxy*, were elaborated in Zhao et al. (2023). The GenBank accession numbers of DNA regions sequenced in this study are listed in Suppl. material 1.

## ﻿Results

**Figure 1.**
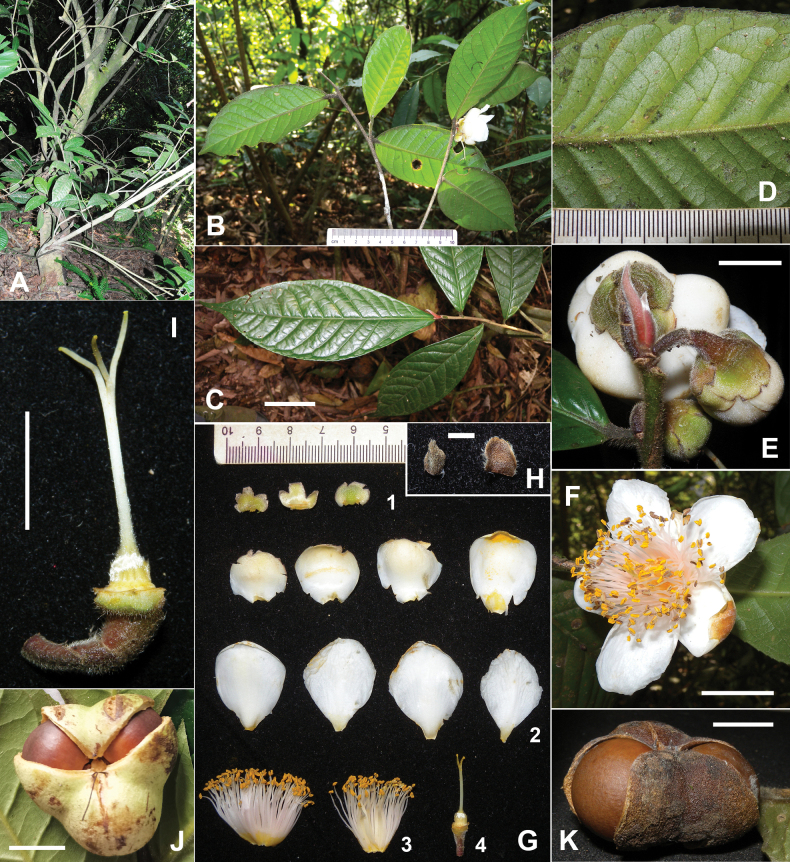
*Camelliayangii* D.Wei Zhao, sp. nov. **A.** Habit; **B, C.** Branchlets; **D.** Abaxial surface of leaf; **E.** Flower buds; **F.** Flower; **G.** A dissected flower without bracteoles, 1-sepals, 2-petals, 3-androecium, 4-pedicel, receptacle and gynoecium; **H.** Bracteoles; **I.** Pedicel, receptacle and gynoecium of a flower; **J, K.** Capsule. Scale bars: 5 cm (**C**); 1 cm (**E**, **I**, **J**, **K**); 2 cm (**F**); 2 mm (**H**). Photos: Zhao D.W. (**A**); Yang S.X. (**B–K**).

**Figure 2.**
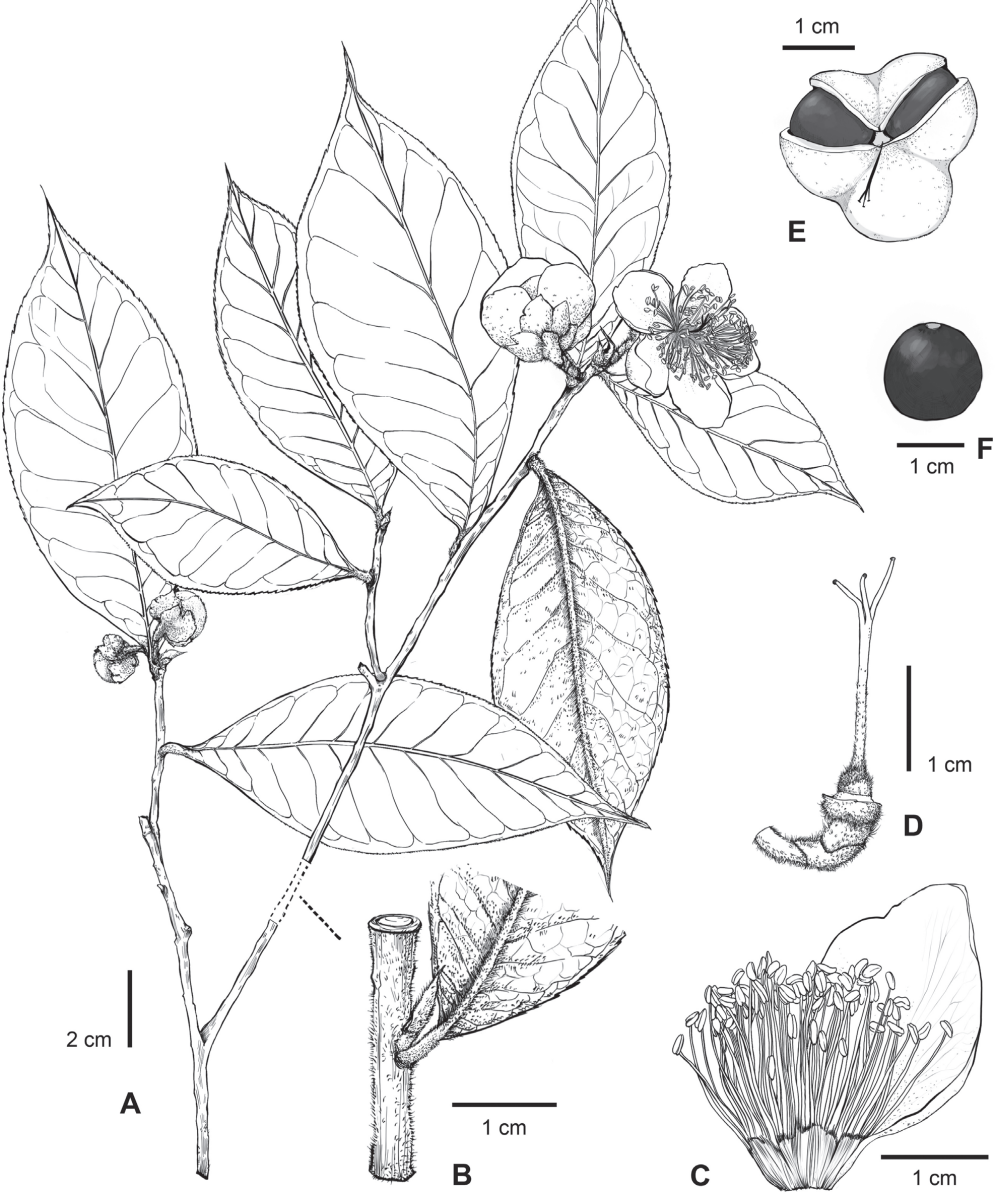
Illustration of *Camelliayangii*. **A.** Branchlet with flowers; **B.** A node of branchlet, showing the indumenta of the branchlet, leaf, and bud; **C.** A part of androecium and a petal; **D.** Pedicel, receptacle and gynoecium of a flower; **E.** Capsule; **F.** Seed. Drawn by Chen M.Q.

The morphology of the new species, *C.yangii* D.Wei Zhao, matches the synapomorphies of species in C.sect.Thea, including bearing a pedicellate flower, two caducous bracteoles, persistent sepals, an apically 3-lobed style, and glabrous seeds (Zhao 2024). The new species is morphologically similar to *C.fangchengensis* S.Ye Liang & Y.C.Zhong and *C.ptilophylla* Hung T.Chang because of the indumenta of vegetative organs. The morphological differences between *C.yangii* and its similar species in C.sect.Thea are listed in Table 1. *Camelliayangii* is characterized by bearing three sepals in the flower (Figs 1, 2), whereas other taxa in C.sect.Thea have five sepals. The new species bears the largest sepals (8–9 × 10–13 mm) in C.sect.Thea (Figs 1, 2, Table 1; Zhao 2024).

**Table 1.** Comparison of *Camelliayangii* and its similar species.

Character	*C.fangchengensis*	*C.longissima*	*C.ptilophylla*	*C.taliensis*	*C.yangii*
Indumentum of new branchlet	densely pubescent	glabrous	densely pubescent	glabrous	densely pubescent
Size of leaf blade	13–29 × 5.5–12.5 cm	7–20 × 3–9 cm	6.5–25 × 3–7.5 cm	7.5–15.5 × 3–6.5 cm	9–21 × 3.5–8.5 cm
Diameter of flower	2–3.5 cm	3–4.5 cm	2.5–3 cm	3–5 cm	4–5.5 cm
Length of pedicel	5–10 mm	17–40 mm	ca. 10 mm	8–15 mm	5–15 mm
Number of sepals	5	5	5	5	3
Size of sepals	2.5–3.5 × 4–5 mm	2.5–5 × 3–6 mm	3–7 × 3.5–7 mm	4–6.5 × 5.5–9 mm	8–9 × 10–13 mm
Indumentum of sepals	abaxially pubescent, adaxially glabrous	abaxially glabrous, adaxially sericeous	abaxially pubescent, adaxially glabrous	abaxially glabrous, adaxially sericeous	abaxially pubescent, adaxially sericeous
Indumentum of ovary	densely pubescent	glabrous	densely pubescent	pubescent	densely pubescent

The natural individuals of *C.yangii* in Malipo County that were found all bore flower buds on 21 November 2023 (*Yang S.X. et al. 7312*–*7315* at KUN, *Zhao D.W. et al. 536[1*]–[*5*] at CSFI), while other taxa of C.sect.Thea that occurred or were planted nearby were flowering or had immature fruits as shown in Table 2. *Camelliayangii* seems to be flowering about one month later than other tea plants (Table 2).

**Table 2.** Observed flowering and fruiting phases of *Camelliayangii* and other taxa of C.sect.Thea occurred or were planted nearby in Yunnan, China in 2023.

Taxon	Locality	Status	Phenology	Vouchers
*C.crispula*	Qiubei	Wild	14 November: flowering	*Zhao et al. 516* (CSFI)
C.crispulavar.multiplex	Wenshan	Wild	22 November: fruiting	*Zhao et al. 539–542* (CSFI)
*C.gymnogyna*	Pingbian	Wild	24 November: fruiting	*Zhao et al. 550* (CSFI)
*C.kwangsiensis*	Guangnan	Wild	7 November: flowering	*Zhao et al. 492(3)*, *(4)*, *(9)*, *(10)* (CSFI)
C.kwangsiensisvar.kwangnanica	Guangnan	Wild	7 November: flowering	*Zhao et al. 492(1)*, *(5)*, *(6)*, *(7)*, *(8)* (CSFI)
C.sinensisvar.assamica	Malipo	Planted	21 November: flowering	*Zhao et al. 534* (CSFI)
C.sinensisvar.pubilimba	Guangnan	Planted	7 November: flowering	*Zhao et al. 491* (CSFI)
*C.tachangensis*	Shizong	Wild	12 November: flowering	*Zhao et al. 508, 509* (CSFI)
*C.yangii*	Malipo	Wild	25 December: flowering	*Yang S.X. & Yin L. 7357–7359* (KUN)

**Figure 3.**
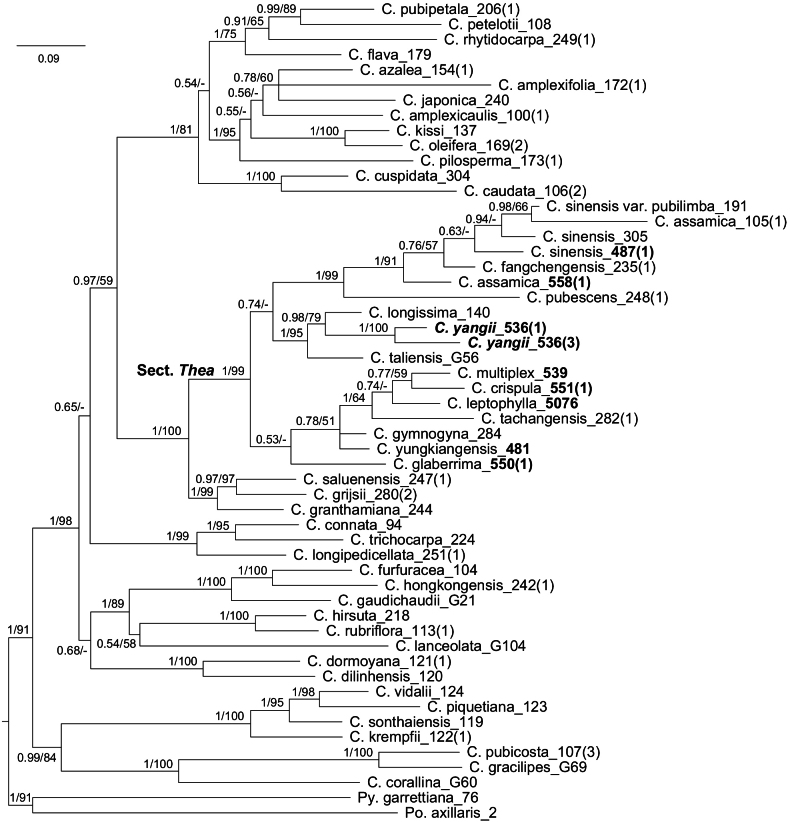
The Bayesian consensus tree reconstructed by the concatenated *RPB2* (introns 11–15 and 23) and *waxy* data for Camelliasect.Thea and related species. Bayesian posterior probabilities (PP) ≥ 0.5 and Bootstrap values (BS; %) ≥ 50 are presented above or below branches as PP/BS. Branch lengths are proportional to the expected nucleotide substitutions per site. Numbers in bold indicate the samples were sequenced in this research.

The DNA sequences of nuclear *RPB2* (introns 11–15 and 23) and *waxy* regions of nine samples, representing eight species in C.sect.Thea, were sequenced in this research (Fig. 3, Suppl. material 1). They and the DNA sequences of 45 samples reported in Zhao et al. (2023) were used to reconstruct a phylogenetic tree of *Camellia* shown in Fig. 3. All 18 samples of C.sect.Thea form a monophyletic group with strong support (Bayesian posterior probability [PP] = 1, Bootstrap [BS, %] = 99). Two samples of *C.yangii* form a strong supported clade (PP = 1, BS = 100) and are nested in the supported monophyletic group (PP = 1, BS = 95) with *C.longissima* and *C.taliensis*. It suggests that the new species, *C.yangii*, is a member of C.sect.Thea and its phylogenetically closely related species are *C.longissima* and *C.taliensis* (Fig. 3). Therefore, the latter two are listed in Table 1 to demonstrate the morphological differences between *C.yangii* and them.

## ﻿Discussion

Many recognized species of C.sect.Thea were described in 1981 and 1984 by Hung T.Chang (Zhao 2024). Except for the basionym of the infraspecific taxa C.tachangensisF.C. Zhangvar.remotiserrata (Hung T.Chang, H.S.Wang & P.S.Wang) T.L.Ming, *C.remotiserrata* Hung T.Chang, H.S.Wang & P.S.Wang that was published in 1990, *C.yangii* is the only new species of natural tea plants described after 40 years. The total number of taxa of C.sect.Thea in the world reaches 12 species and seven infraspecific taxa, and China harbors all of them.

As a supported member of C.sect.Thea (Fig. 3), *C.yangii* may be the hairiest species of tea plants. It is unique in *Camellia* by bearing three large sepals in the flower, whereas other species of the genus generally bear five sepals (Table 1). *Camelliayangii* and its morphologically closely related species, *C.fangchengensis* and *C.ptilophylla*, are nested in the different supported clades within C.sect.Thea (Fig. 3). Meanwhile, the new species can be easily distinguished from its phylogenetically closely related plants, *C.longissima* and *C.taliensis*, by its hairy vegetative organs (vs. generally glabrous for the latter two) (Figs 1, 2, Table 1). It would be unreasonable to infer the phylogenetic relationships among tea plants solely based on the variation of morphological characters as Chang (1981b) and Ming (2000) had done.

The flowering phase of *C.yangii* is later than those of other tea plants nearby (Table 2). The distinct flowering periods may not suggest that the new species can naturally hybridize with other taxa of C.sect.Thea, which will support its novelty based on the biological species concept (Butlin and Stankowski 2020). Together with the morphological (Table 1) and phylogenetic (Fig. 3) analyses presented above based on the phenetic species concept (Sokal and Crovello 1970) and phylogenetic species concept (Mishler and Brandon 1987), respectively, it is reasonable to conclude that *C.yangii* is a new species of C.sect.Thea.

*Camelliayangii* has a red or purplish red and densely pubescent terminal bud (Fig. 1C, E), which can be suggested as a rare germplasm resource of tea plants based on the Chinese Agricultural Standard (NY/T 2031-2011). However, the top priority for the new species should be conservation. *Camelliayangii* is only known from a single population with fewer than 10 individuals in the tropical montane forest. It is extremely vulnerable to overexploitation or deforestation. Therefore, its detailed locality is absent here for conservation reasons. Further field surveys and ex-situ asexual propagations are urgently needed for *C.yangii* to avoid extinction.

### ﻿Taxonomic treatment

#### ﻿ Camellia yangii

Taxon classificationPlantaeEricalesTheaceae

D.Wei Zhao sp. nov.

3C9435AB-4CB2-5BB5-B1C2-07E221589DB8

urn:lsid:ipni.org:names:77363261-1

##### Type material.

***Holotype***: China • Yunnan: Malipo, in evergreen montane forest, 858 m, 25 December 2023, *Yang S.X. & Yin L. 7357* (holotype: KUN 1628256!; isotypes: CSFI!, KUN 1628257!, KUN 1628258!)

##### Diagnosis.

Similar to *C.fangchengensis* and *C.ptilophylla* by the densely pubescent new branchlets, abaxial surface of leaves and pedicel, but differs from them in bearing a larger flower (4–5.5 cm in diam. vs. 2–3.5 cm in diam.), less (3 vs. 5) but larger (8–9 × 10–13 mm vs. 2.5–7 × 3.5–7 mm) sepals, and sericeous (vs. glabrous) adaxial surface of the sepals (Table 1).

##### Description.

Evergreen shrubs or trees, 5–8 m tall. ***Bark*** grayish yellow. ***New branchlets*** densely pubescent, ***terminal buds*** red or purplish red, densely pubescent. ***Petioles*** 3–5 mm long, densely pubescent; ***leaf blades*** elliptic, oblong, or obovate, 9–21 × 3.5–8.5 cm, coriaceous, abaxially yellowish green, densely pubescent, adaxially dark green, glabrous or puberulous along midrib at base, midrib and secondary veins abaxially elevated and adaxially impressed, secondary veins 10–13 pairs, base cuneate, margin serrulate, apex acuminate. ***Flowers*** axillary, solitary or paired, 4–5.5 cm in diam. ***Pedicel*** 5–15 mm long, densely pubescent. ***Bracteoles*** 2, caducous, ovate, 2–3 × 2–2.5 mm, abaxially pubescent, adaxially glabrous. ***Sepals*** 3, persistent, suborbicular, 8–9 × 10–13 mm, abaxially pubescent, adaxially sericeous, margin ciliolate. ***Petals*** 7–8 in 1–2 whorls, white, or the outmost petal green at apex, elliptic to obovate, 15–35 × 15–25 mm, outer petals pubescent or puberulous on both surfaces, inner petals puberulous at base or glabrous on both surfaces, apex obtuse to rounded, inner 4–5 petals basally adnate to filament whorl for 2–4 mm. ***Stamens*** numerous, 20–25 mm long; ***filaments*** white or slightly pink, glabrous, outer filaments basally connate for 3–5 mm. ***Ovary*** globose to ovoid, densely pubescent. ***Styles*** 1, 18–22 mm long, gradually becoming glabrous upwards, apically 3-lobed for 5–8 mm. ***Capsule*** tri-coccal, ca. 3.5 cm in diam., ca. 2 cm in height, 3-loculed with 1 seed per locule; pericarp 1–2 mm thick. ***Seeds*** fuscous, globose, ca. 1.5 cm in diam., glabrous, Figs 1, 2.

##### Phenology.

Flowering December, fruiting August–September (Table 2).

##### Paratypes.

China. • Yunnan: Malipo County, in evergreen montane forest, 858 m, August 2023, *Yang S.X. & Xiao B. 7122* (KUN); same place, 21 November 2023, *Yang S.X. et al. 7312*–*7315* (KUN, equal to *Zhao D.W. et al. 536[1*]–[*4*] at CSFI, respectively), *Zhao D.W. et al. 536(5)* (CSFI); same place, 25 December 2023, *Yang S.X. & Yin L. 7358* (KUN), *7359* (KUN).

##### Distribution and habitat.

*Camelliayangii* is endemic to the tropical evergreen montane forest in Malipo County.

##### Etymology.

*Camelliayangii* is named after the leading collector of its type, Dr. Shixiong Yang, an expert of the family Theaceae at Kunming Institute of Botany, Chinese Academy of Sciences. The Chinese name of *C.yangii* is proposed as ”三萼茶” because it bears three sepals in the flower.

## Supplementary Material

XML Treatment for Camellia yangii

## ﻿Additional information

### Author ORCIDs

Dongwei Zhao https://orcid.org/0000-0002-7761-7127

### Data availability

All of the data that support the findings of this study are available in the main text and GenBank as shown in Suppl. material 1.

## Supplementary materials

Supplementary material 1 Samples used in the research Data type xlsx https://binary.pensoft.net/file/1352623This dataset is made available under the Open Database License (http://opendatacommons.org/licenses/odbl/1.0/). The Open Database License (ODbL) is a license agreement intended to allow users to freely share, modify, and use this Dataset while maintaining this same freedom for others, provided that the original source and author(s) are credited.Author: Dongwei Zhao
